# Pseudocirrhosis due to liver metastasis from lung adenocarcinoma

**DOI:** 10.1111/1759-7714.14084

**Published:** 2021-07-17

**Authors:** Naoki Shijubou, Toshiyuki Sumi, Yoshiko Keira, Hideaki Shiraishi, Yuta Nagahisa, Keigo Matsuura, Motoki Sekikawa, Yuichi Yamada, Hisashi Nakata, Hirofumi Chiba

**Affiliations:** ^1^ Department of Pulmonary Medicine Hakodate Goryoukaku Hospital Hakodate Japan; ^2^ Department of Respiratory Medicine and Allergology Sapporo Medical University School of Medicine Sapporo Japan; ^3^ Department of Surgical Pathology Hakodate Goryoukaku Hospital Hakodate Japan; ^4^ Department of Pulmonary Medicine Mitsui Memorial Hospital Tokyo Japan

**Keywords:** adenocarcinoma, liver metastasis, lung cancer, pseudocirrhosis

## Abstract

Pseudocirrhosis is a radiological diagnosis of cirrhosis without histological evidence and occurs as a complication of liver metastases from solid tumors. A 50‐year‐old man without any previous history of liver disease was diagnosed with adenocarcinoma of the left upper lung lobe and liver metastasis. After chemotherapy, the liver metastases shrank; however, over time, the liver shrank and showed cirrhosis‐like morphological changes. His performance status deteriorated due to ascites and leg edema, and chemotherapy was terminated. Physicians treating lung adenocarcinoma with liver metastases should be aware that pseudocirrhosis is a rare but important complication that can worsen performance status (PS) and hinder treatment continuation.

## Graphical abstract

Liver metastasis from adenocarcinoma of the lung with abundant stromal growths showed cirrhosis‐like morphological changes in the course of treatment, ascites and marked leg edema. Pseudocirrhosis due to liver metastasis from lung adenocarcinoma is a rare but important complication that can worsen performance status and hinder treatment continuation.

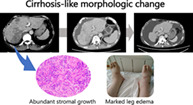

## INTRODUCTION

Pseudocirrhosis is a radiological diagnosis of liver cirrhosis with no histological evidence. It is characterized by morphological changes similar to those in cirrhosis, such as retraction of the capsule, nodule formation, parenchymal atrophy, and enlargement of the caudate lobe.^1^, ^2^ Pseudocirrhosis has been previously reported in cases of solid tumors, mainly breast cancer.^3^ Regarding lung cancer, one case of pseudocirrhosis in a patient with small‐cell lung cancer has recently been reported;^4^ however, pseudocirrhosis has not been previously reported in the context of lung adenocarcinoma. Herein, we report a case of lung adenocarcinoma associated with pseudocirrhosis during chemotherapy.

## CASE REPORT

**FIGURE 1:**
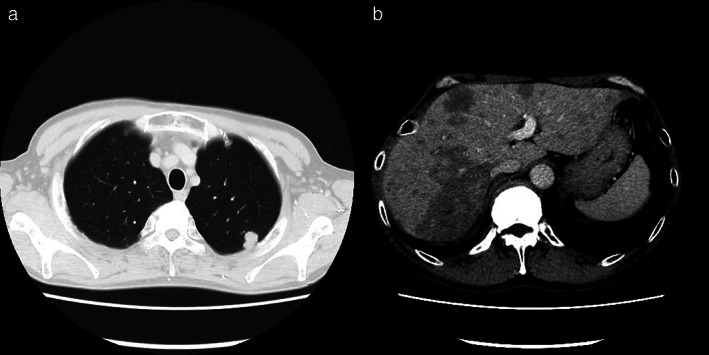
Pretreatment computed tomography (CT) findings. (a) A nodular shadow in the left upper lobe. (b) Multiple low‐density areas in the liver

**FIGURE 2:**
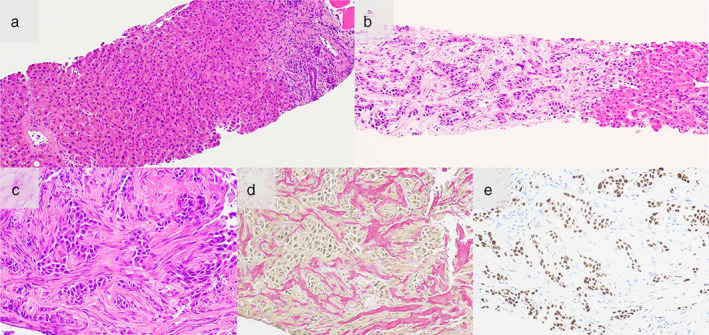
Histopathological findings of the biopsy specimen obtained from one of the liver tumors. (a) The background liver is structurally preserved, with no evidence of periportal, central venous, or stem cell fibrosis (hematoxylin and eosin staining [HE staining], × 10). (b) In the region involving the border with the background liver, fibrous stroma is observed only in the area of cancer growth on the left side (HE staining, × 10). (c) Abundant stromal growth is observed between vesicular tumor cells (HE staining, × 20). (d) Red‐stained regions indicate collagen‐rich fibers (Elastica van Gieson stain × 20). (e) The nuclei of the tumor cells are positive for thyroid transcription factor‐1 (TTF1) (× 20)

**FIGURE 3:**
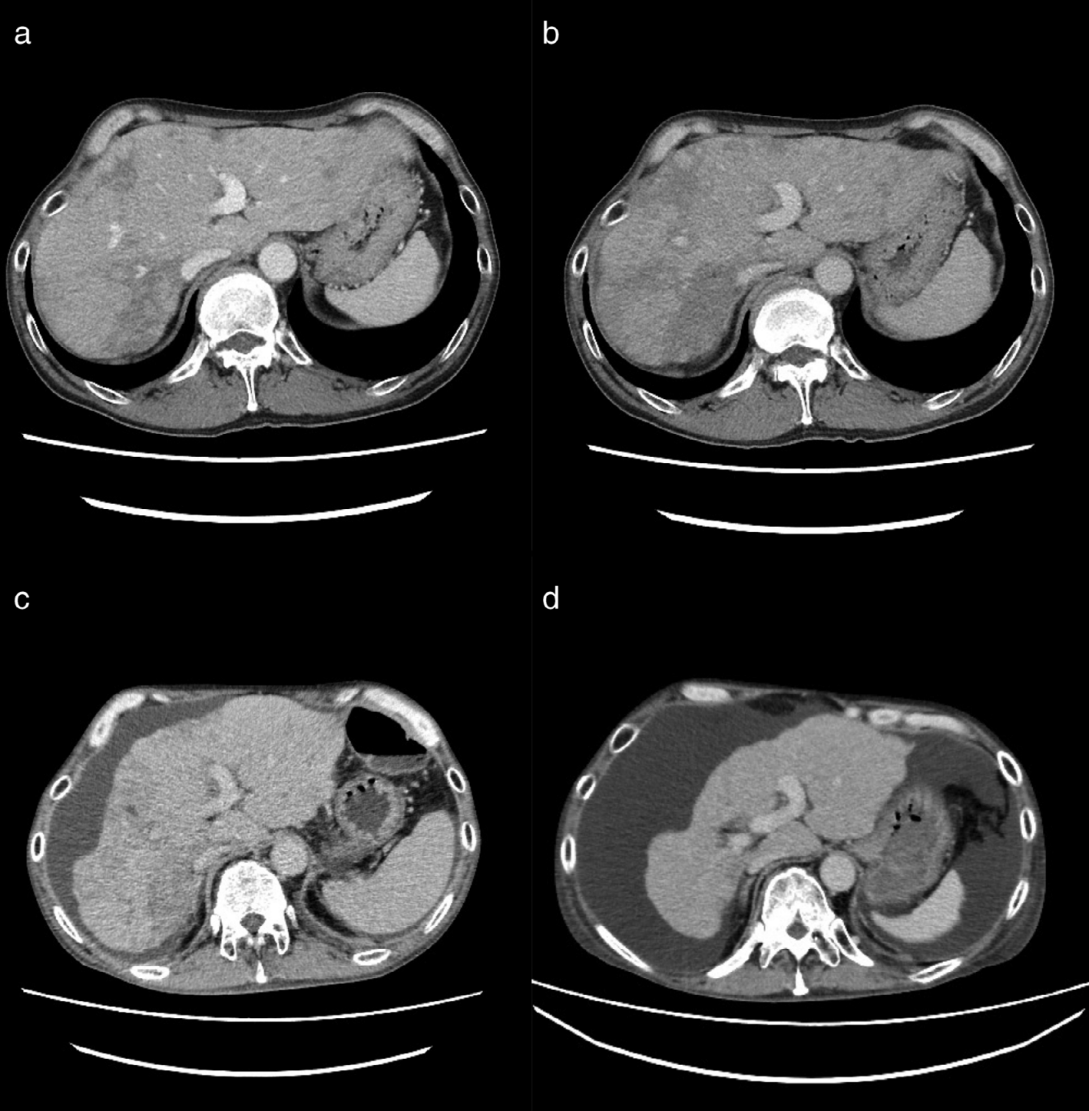
Computed tomography (CT) findings of liver metastasis and liver morphology over time. (a) Three months after the first visit, a decrease in the low‐density area in the tumor was noted in response to first‐line treatment. (b) Five months after the first visit, during maintenance therapy, an increase in the low‐density area was observed, and second‐line therapy was initiated. (c) Seven months after the first visit, the patient responded to second‐line therapy, and a decrease in the low‐density area of the tumor was observed; however, ascites appeared, followed by liver shrinkage. (d) Nine months after the first visit, a further increase was observed in ascites, the liver showed cirrhosis‐like morphology, and the patient's performance status worsened

**FIGURE 4:**
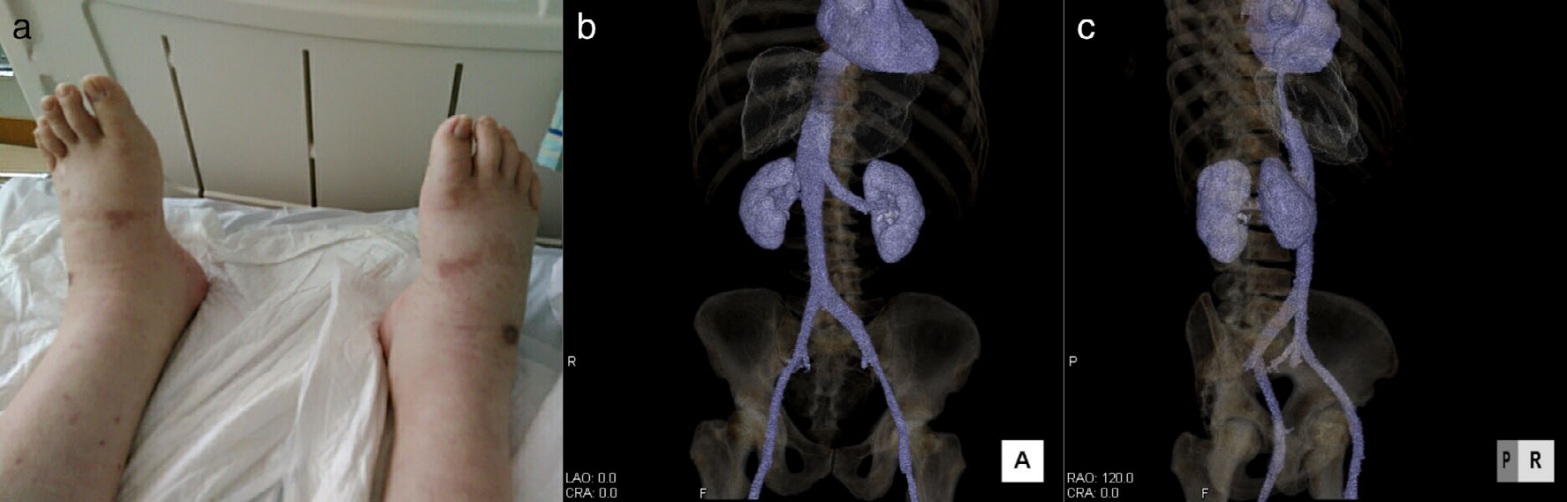
Symptoms associated with pseudocirrhosis and three‐dimensional computed tomography (CT) findings of the inferior vena cava. (a) Marked leg edema; (b) and (c) Stenosis of the inferior vena cava near the liver

A 50‐year‐old man with no history of alcohol consumption, liver disease, or hepatitis virus infection was referred to our institution because of abnormal shadows and a markedly elevated carcinoembryonic antigen (CEA) level of 36 162 ng/ml (normal, <5 ng/ml). Computed tomography (CT) showed a 20‐mm lung nodule in the left upper lobe and multiple liver tumors (Figure 1), and a biopsy of one of the liver tumors revealed metastasis from lung adenocarcinoma (Figure 2). The patient was diagnosed with adenocarcinoma of the left upper lung lobe and liver metastasis, cT2bN3M1c, stage IVB. There were no driver mutations, and programmed death‐ligand 1 tumor proportion score was 1%. The patient received chemotherapy comprising carboplatin (area under the curve, 6), paclitaxel (200 mg/m^2^), bevacizumab (15 mg/kg), and atezolizumab (1200 mg/bodyweight) every three weeks. After four cycles of chemotherapy, the primary tumor and liver metastasis had shrunk; however, during maintenance therapy with bevacizumab and atezolizumab, only the hepatic metastases grew, and the size of the primary tumor reduced from 20 to 10 mm. Thus, the patient was treated with docetaxel and ramucirumab as second‐line therapy. His CEA level decreased to 21 419 ng/ml, and liver dysfunction was alleviated before and after the second‐line therapy (alanine aminotransferase, 174 to 49 IU/L; aspartate aminotransferase, 81 to 38 IU/L; alkaline phosphatase, 3096 to 1326 IU/L; and γ‐glutamyl transpeptidase, 1769 to 508 IU/L). Low‐density areas on the CT appeared to have shrunk; however, abdominal CT showed ascites; the liver shrank over time, indicating cirrhosis‐like morphological changes (Figure 3). Ascites cytology showed no malignancy; therefore, his condition was diagnosed as pseudocirrhosis due to liver metastasis from lung adenocarcinoma. The patient had marked edema of the lower leg and stenosis of the inferior vena cava near the liver (Figure 4). An inferior vena cava stent was inserted, and the patient's leg edema was temporarily relieved; however, his performance status (PS) gradually worsened. At worst, the Child‐Pugh score was 9 points, Class B. (total bilirubin 1; serum albumin 3; prothrombin time 1; ascites 3; hepatic encephalopathy 1) The patient was referred to a palliative care hospital, and chemotherapy was discontinued. The patient died 10 months after first‐line treatment had been initiated.

## DISCUSSION

We encountered a case of pseudocirrhosis due to liver metastasis from lung adenocarcinoma. To our knowledge, this is the first report of pseudocirrhosis in a patient with lung adenocarcinoma.

The causes of pseudocirrhosis include regression of the liver capsule owing to chemotherapy and the desmoplastic response to invasive tumors.^5^, ^6^ Therefore, it would be difficult to predict and prevent the incidence/progression of pseudocirrhosis. In this case, the patient's CEA level decreased, and CT showed shrinkage of the low‐density areas, especially after second‐line therapy, suggesting the influence of scar contraction of the liver tumor owing to the response to chemotherapy. In contrast, because the CEA levels remained relatively high even after reduction and cancer stroma with abundant fibrosis was noted during pathological diagnosis, the cirrhosis‐like morphological changes may have been influenced by the cancer stroma. Since no low absorption area was observed around the inferior vena cava on CT, which would have suggested liver metastasis, the inferior vena cava obstruction may be due to secondary liver fibrosis. Although immune checkpoint inhibitors (ICIs) were similarly administered in this case, cirrhosis‐like changes are not considered immune‐related adverse events because chronic hepatitis and liver cirrhosis are generally absent in patients with ICI‐induced liver toxicity.^7^ Pseudocirrhosis may lead to complications associated with cirrhosis (ascites, splenomegaly, and esophageal varices, among others), and symptomatic treatment for these complications is necessary.^2^, ^5^ In this case, we tried to improve the symptoms by performing abdominal puncture as appropriate.

In conclusion, our case shows that pseudocirrhosis and associated ascites, leg edema, and inferior vena cava stenosis can occur in patients with lung adenocarcinoma. Clinicians treating lung adenocarcinoma with liver metastases should be aware that pseudocirrhosis is a rare but important complication that can reduce PS and hinder treatment continuation.

## CONFLICT OF INTEREST

The authors declare that they have no conflicts of interest.
